# Does bipolar disorder differ from other mental illnesses in terms of emotion dysregulation? A systematic review and meta-analysis

**DOI:** 10.1192/j.eurpsy.2023.1199

**Published:** 2023-07-19

**Authors:** M. De Prisco, V. Oliva, G. Fico, J. Radua, I. Grande, N. Roberto, G. Anmella, D. Hidalgo-Mazzei, M. Fornaro, A. de Bartolomeis, A. Serretti, E. Vieta, A. Murru

**Affiliations:** 1 Bipolar and Depressive Disorders Unit, Institute of Neuroscience, Hospital Clinic, University of Barcelona, IDIBAPS, CIBERSAM, Barcelona, Spain; 2 Section of Psychiatry, Department of Neuroscience, Reproductive Science and Odontostomatology, Federico II University of Naples, Naples; 3 Department of Biomedical and Neuromotor Sciences, University of Bologna, Bologna, Italy; 4 Imaging of Mood- and Anxiety-Related Disorders (IMARD) Group, IDIBAPS, Barcelona; 5 CIBERSAM, Instituto de Salud Carlos III, Madrid, Spain; 6 Early Psychosis: Interventions and Clinical-Detection Lab, Institute of Psychiatry, Psychology & Neuroscience, King’s College London, London, United Kingdom; 7 Centre for Psychiatric Research and Education, Department of Clinical Neuroscience, Karolinska Institutet, Stockholm, Sweden

## Abstract

**Introduction:**

Emotion regulation (ER) is the ability to assess, monitor, or modify emotional reactions to achieve a goal (Gross. Psychological inquiry 2015; 26 1-26). When ER strategies are rigidly or maladaptively applied, emotional dysregulation (ED) can occur (Thompson. Development and psychopathology 2019; 31 805-815). ED is common in people diagnosed with bipolar disorder (BD), but it can also be described in other clinical populations given its transdiagnostic nature. Numerous aspects of ED have been described in BD (De Prisco *et al*. Neuroscience & Biobehavioral Reviews 2022; 104914), but it is unclear whether these manifest similarly in other conditions such as major depressive disorder (MDD) or borderline personality disorder (BPD), or whether they are specific to BD.

**Objectives:**

The objective of this systematic review and meta-analysis is to examine the literature comparing BD with other psychiatric disorders in terms of ED, focusing on those studies using validated clinical tools.

**Methods:**

A systematic search from inception to April 28th, 2022, was conducted exploring the PubMed/MEDLINE,EMBASE, Scopus, and PsycINFO databases. Those studies providing quantitative data on ED in people diagnosed with BD and compared with clinical groups were eligible for inclusion. No restriction about age, sample size, or language were applied. Random effect meta-analyses were conducted, and effect sizes were calculated as standardized mean differences (SMD).

**Results:**

A total of 3,239 records was identified and, after duplicate removal and title/abstract evaluation, 112 were explored at the full text. Twenty-nine studies were finally included, and it was possible to perform a meta-analysis with twenty-two (145 comparisons) of them. Only studies comparing BD with MDD, and BPD provided sufficient data to perform a meta-analysis. People with BD did not differ from people with MDD in most of the comparisons considered. However, BD patients presented higher positive rumination (two comparisons: SMD=0.46; CI=0.27, 0.64; p=8.5e-07; I^2^=0%; and SMD=0.34; CI=0.15, 0.52; p=2.7e-04; I^2^=0%) and risk-taking behaviors (SMD=0.48; CI=0.27, 0.69; p=8.11e-06; I^2^=0%). In contrast, people with BPD displayed an overall higher degree of ED (SMD=-1.22; CI=-1.94, -0.5; p=9.1e-04; I^2^= 90.7) and used fewer adaptive ER strategies. Additionally, higher levels of self-blaming (SMD=-0.80; CI=-1.11, -0.50; p=2.68e-07; I^2^=0) and impulsive behavior (SMD=-0.76; CI=-0.89, -0.63; p=5.4e-29; I^2^=0) were observed.

**Image:**

**Figure:**
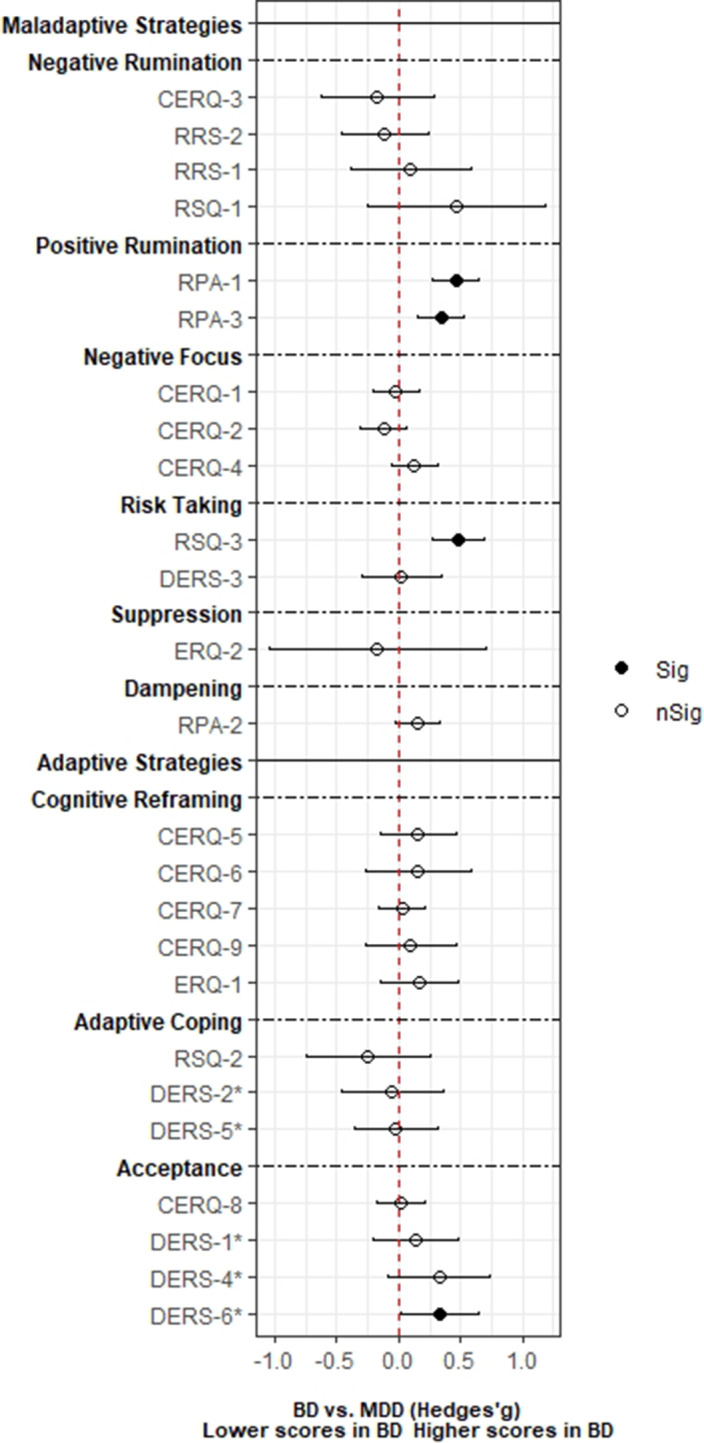

**Image 2:**

**Figure:**
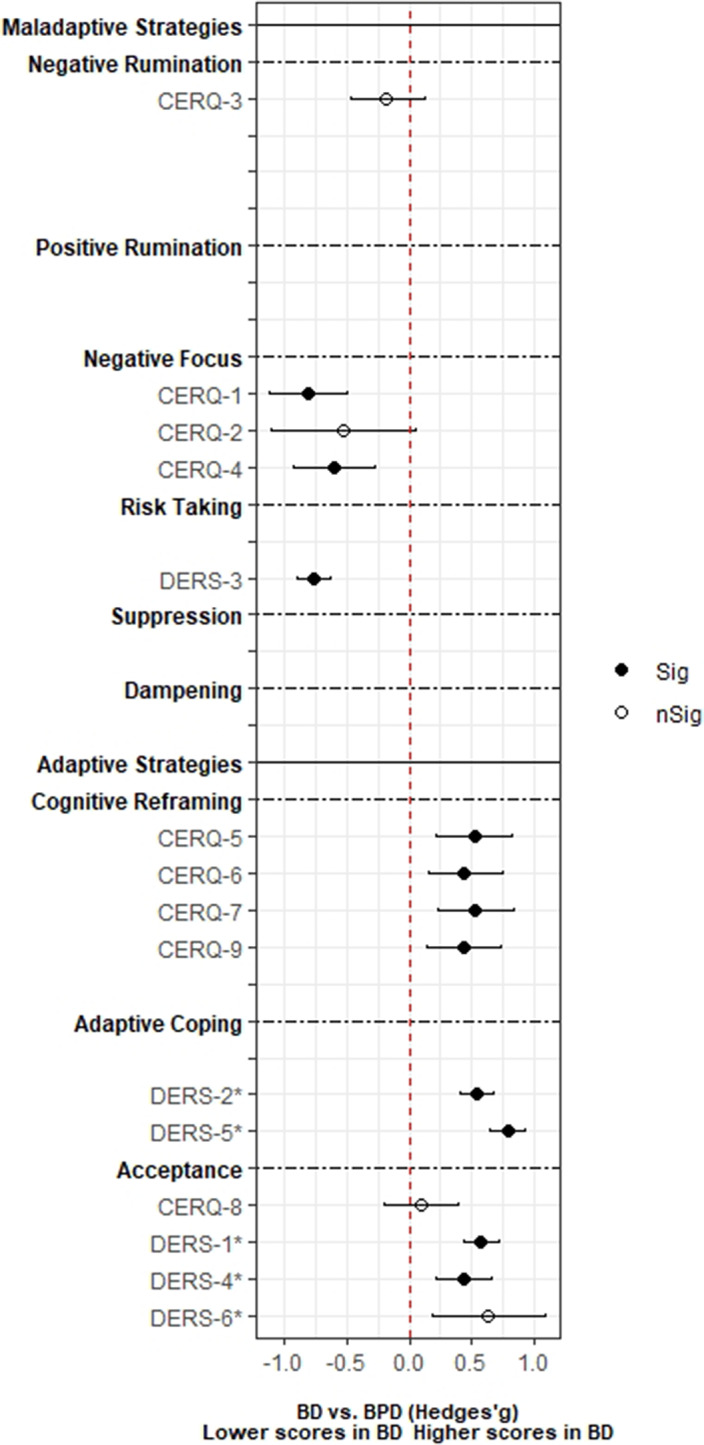

**Conclusions:**

ED is a trans-diagnostic construct that spans a continuum of different psychiatric disorders. Outlining the specific clinical features of one disorder versus another may help future research to increase our knowledge of these issues and develop new treatment strategies to reduce the clinical burden of these patients.

**Disclosure of Interest:**

M. De Prisco: None Declared, V. Oliva: None Declared, G. Fico Grant / Research support from: “La Caixa” Foundation (ID 100010434 - fellowship code LCF/BQ/DR21/11880019), Consultant of: Angelini, Janssen-Cilag and Lundbeck, J. Radua Grant / Research support from: Spanish Ministry of Science and Innovation (PI19/00394, CPII19/00009) integrated into the Plan Nacional de I+D+I and co-financed by the ISCIII-Subdirección General de Evaluación and the Fondo Europeo de Desarrollo Regional (FEDER) and the Instituto de Salud Carlos III, I. Grande Grant / Research support from: Spanish Ministry of Science and Innovation (MCIN) (PI19/00954) integrated into the Plan Nacional de I+D+I and cofinanced by the ISCIII-Subdirección General de Evaluación y el Fondos Europeos de la Unión Europea (FEDER, FSE, Next Generation EU/Plan de Recuperación Transformación y Resiliencia_PRTR ); the Instituto de Salud Carlos III; the CIBER of Mental Health (CIBERSAM); and the the Secretaria d’Universitats i Recerca del Departament d’Economia i Coneixement (2017 SGR 1365), CERCA Programme / Generalitat de Catalunya as well as the Fundació Clínic per la Recerca Biomèdica (Pons Bartran 2022-FRCB_PB1_2022), Consultant of: ADAMED, Angelini, Casen Recordati, Ferrer, Janssen Cilag, and Lundbeck, Lundbeck-Otsuka, Luye, SEI Healthcare, N. Roberto: None Declared, G. Anmella Grant / Research support from: Rio Hortega 2021 grant (CM21/00017) from the Spanish Ministry of Health financed by the Instituto de Salud Carlos III (ISCIII) and co-financed by the Fondo Social Europeo Plus (FSE+), Consultant of: Janssen-Cilag, Lundbeck, Lundbeck/Otsuka, and Angelini, D. Hidalgo-Mazzei Grant / Research support from: Juan Rodés JR18/00021 granted by the Instituto de Salud Carlos III (ISCIII), M. Fornaro: None Declared, A. de Bartolomeis Consultant of: Janssen, Lundbeck, and Otsuka and lecture fees for educational meeting from Chiesi, Lundbeck, Roche, Sunovion, Vitria, Recordati, Angelini and Takeda; he has served on advisory boards for Eli Lilly, Jansen, Lundbeck, Otsuka, Roche, and Takeda, Chiesi, Recordati, Angelini, Vitria, A. Serretti Consultant of: Abbott, Abbvie, Angelini, AstraZeneca, Clinical Data, Boehringer, Bristol-Myers Squibb, Eli Lilly, GlaxoSmithKline, Innovapharma, Italfarmaco, Janssen, Lundbeck, Naurex, Pfizer, Polifarma, Sanofi, Servier, and Taliaz, E. Vieta Grant / Research support from: Spanish Ministry of Science and Innovation (PI18/00805, PI21/00787) integrated into the Plan Nacional de I+D+I and co-financed by the ISCIII-Subdirección General de Evaluación and the Fondo Europeo de Desarrollo Regional (FEDER); the Instituto de Salud Carlos III; the CIBER of Mental Health (CIBERSAM); the Secretaria d’Universitats i Recerca del Departament d’Economia i Coneixement (2017 SGR 1365), the CERCA Programme, and the Departament de Salut de la Generalitat de Catalunya for the PERIS grant SLT006/17/00357. Thanks the support of the European Union Horizon 2020 research and innovation program (EU.3.1.1. Understanding health, wellbeing and disease: Grant No 754907 and EU.3.1.3. Treating and managing disease: Grant No 945151), Consultant of: AB-Biotics, AbbVie, Angelini, Biogen, Boehringer-Ingelheim, Celon Pharma, Dainippon Sumitomo Pharma, Ethypharm, Ferrer, Gedeon Richter, GH Research, Glaxo-Smith Kline, Janssen, Lundbeck, Medincell, Novartis, Orion Corporation, Organon, Otsuka, Rovi, Sage, Sanofi-Aventis, Sunovion, Takeda, and Viatris, A. Murru Grant / Research support from: Spanish Ministry of Science and Innovation (PI19/00672) integrated into the Plan Nacional de I+D+I and co-financed by the ISCIII-Subdirección General de Evaluación and the Fondo Europeo de Desarrollo Regional (FEDER), Consultant of: Angelini, Idorsia, Lundbeck, Pfizer, Takeda

